# Impact Assessment of the M_s_7.0 Earthquake on Jiuzhaigou Valley from the Perspective of Vegetation Net Primary Productivity

**DOI:** 10.3390/s22228875

**Published:** 2022-11-16

**Authors:** Chenyuan Wang, Xudong Hu, Kaiheng Hu, Shuang Liu, Wei Zhong

**Affiliations:** 1 Hubei Key Laboratory of Disaster Prevention and Mitigation, College of Civil Engineering and Architecture, China Three Gorges University, Yichang 443002, China; 2 College of Civil Engineering and Architecture, China Three Gorges University, Yichang 443002, China; 3 Key Laboratory of Mountain Hazards and Earth Surface Processes, Chinese Academy of Sciences, Chengdu 610041, China; 4 Institute of Mountain Hazards and Environment, Chinese Academy of Sciences, Chengdu 610041, China

**Keywords:** net primary productivity, Jiuzhaigou earthquake, CASA model, geological hazard, seismic intensity

## Abstract

In order to assess the impact of the M_s_7.0 Jiuzhaigou earthquake that occurred on 8 August 2017 on vegetation, the Carnegie-Ames-Stanford Approach (CASA) model was adopted to estimate the vegetation net primary productivity (NPP) of Jiuzhaigou Valley, one of the World Heritage Sites, in July, August and September from 2015 to 2019. Then the characteristics of the impact of different earthquake-induced geohazards on vegetation were discussed, and a vulnerability-resilience assessment system concerning the seismic intensity was proposed. The results show that the NPP_max_ and NPP_mean_ values in Jiuzhaigou Valley first decreased and then increased and were 151.5–261.9 gC/m^2^ and 54.6–116.3 gC/m^2^, respectively. The NPP value of more than 70% area was 90–150 gC/m^2^ in July. In August, the NPP_mean_ values decreased, and the areas with lower values became larger; the NPP_mean_ values of most areas affected by geohazards were 60–150 gC/m^2^. During the earthquake, the NPP_mean_ values of areas hit by geohazards sharply declined by 27.2% (landslide), 22.4% (debris flow) and 15.7% (collapse) compared with those in the same month in 2016. Vegetation in debris flow zones showed a stronger recovery, with a maximum NPP value increase of about 23.0% in September 2017. The vegetation gradually recovered after the earthquake, as indicated by the uptrend of the NPP values in the corresponding period in 2018 and 2019. In general, the reduction magnitude of NPP values decreased year by year in comparison to that in 2015 and 2016, and the decrease slowed down after the earthquake. The vulnerability and resilience index corresponding to the three seismic intensity ranges were 0.470–0.669 and 0.642–0.693, respectively, and those of Jiuzhaigou Valley were 0.473 and 0.671, respectively. The impact coefficient defined to represent the impact of the earthquake on NPP was 0.146–0.213. This paper provides a theoretical reference and guidance for the impact assessment of earthquakes on the ecosystem.

## 1. Introduction

Net primary productivity (NPP), an important metric of ecosystem growth, is greatly affected by natural events (such as earthquakes, geohazards, and climate change) and human activities [1]. NPP was adopted as a key indicator of vegetation coverage and the effect of natural disasters and human activities on vegetation restoration [2,3,4]. Jiuzhaigou Valley was listed as a World Heritage Site (WHS) in 1992 by UNESCO [5] and is known as one of the most famous national parks in China. Unfortunately, an M_s_7.0 earthquake at 33.20° N, 103.82° E struck Jiuzhaigou Valley on 8 August 2017. The strong earthquake and geohazards induced by it, such as landslides, debris flows, and avalanches, seriously damaged the ecosystem and affected over 30% of vegetation there [6]. The trend of vegetation change, which can be represented by the vegetation NPP, provides important information for landscape protection and restoration at WHSs after earthquakes [7,8].

Accurate estimation of terrestrial vegetation NPP is difficult due to the complex effects of various factors. With the development of the terrestrial ecosystem model (TEM) [9], the equilibrium terrestrial biosphere model (BIOME3) [10], the BioGeochemical Cycles (BIOME-BGC) model [11], the carbon exchange between vegetation, soil and atmosphere (CEVSA) model [12], and the ecosystem modeling framework LPJ-GUESS [13], the estimation accuracy was greatly improved, which is more suitable in small areas than in large-scale regions [14]. Although these models were well known for simplicity and fewer parameter requirements, they are limited by the uncertainty in iterations, image inaccuracy, heterogeneous vegetation, standard errors of measurements, and phenological characteristics. In the context of global advances in 3S technologies, simulation models for large-scale NPP estimation were developed [15,16]. The Carnegie-Ames-Stanford Approach (CASA) model is a global NPP simulation model combining multi-year satellite, climate, and other land surface remote-sensing databases. Many researchers have applied this model in studies related to ecosystems at high altitudes [17,18,19]. The advantages mentioned above have promoted its application in the assessment of the relationship between the dynamic changes in NPP and the driving forces. For instance, the NPP changing trend was related to urbanization [20], climate change [2], ecological frangibility [21], and human activities. Li et al. [22] estimated the NPP in the disaster areas by RS and GIS technologies before and after the 2008 M_s_8.0 Wenchuan earthquake and found that the earthquake and the geohazards induced by it had a more obvious impact on the vegetation NPP in a short period. Although there were many studies on the relationship between NPP and climate change or human activities, the response of NPP to earthquakes has not been extensively investigated.

In this study, the data of three types of geohazards induced by the earthquake in Jiuzhaigou Valley were integrated to analyze the NPP response according to the following procedures:

(1) The distribution and characteristics of NPP before and after the earthquake estimated by the CASA model were analyzed.
(2) Based on the NPP results above, earthquake-induced geohazards (coseismic landslide, collapse and debris flow) identified by field survey were considered for grasping the dynamic NPP changes caused by earthquake-induced geohazards. The features of the impact of each type of geohazard on the ecosystem were preliminarily discussed.
(3) In accordance with the assessment methods of Hu et al. [6], the assessment system concerning seismic intensity was constructed for quantificational analysis of the impact of the earthquake on vegetation NPP in Jiuzhaigou Valley.

## 2. Study Region and Methodology

**Figure 1:**
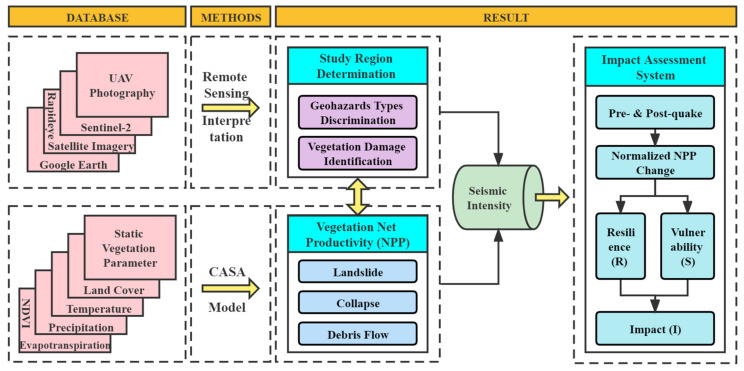
Workflow of the research.

The study region was determined, and the earthquake-induced geohazards were identified through field surveys. In addition, relevant data were integrated, the estimation method and the impact factors were determined. The workflow of the research is presented in Figure 1.

### 2.1. Study Region

#### 2.1.1. Overview of Jiuzhaigou Valley

**Figure 2:**
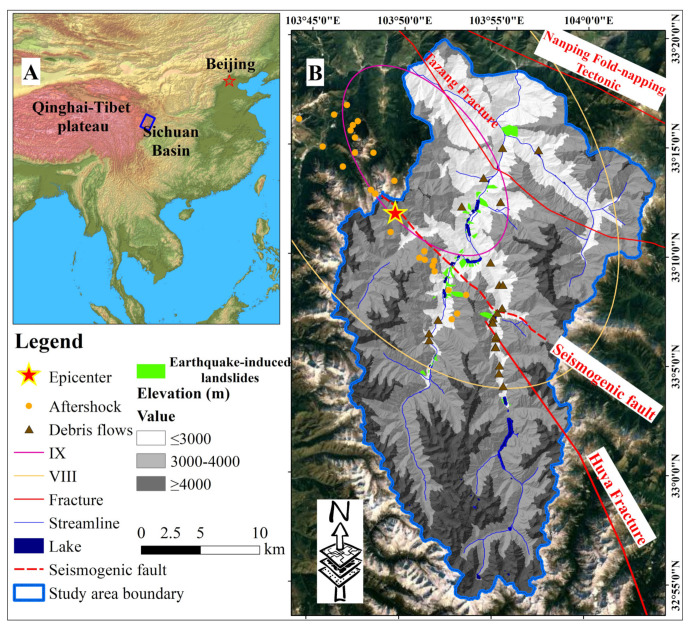
Geomorphological and geological settings of the study region. (**A**) Location of study region; (**B**) distribution of elevation and earthquake-induced geohazards in the park; the base map with a spatial resolution of 16 × 16 m was downloaded from Google earth, and the spatial resolution of DEM is 10 m × 10 m. “IX” and “VIII” refer to seismic intensity VIII and IX, respectively.

The Jiuzhaigou park is located in the transition zone of the western margin of the Sichuan Basin and the Qinghai–Tibet Plateau, with an area of approximately 720 km^2^ and a vegetation coverage rate of 85.5%. The vegetation types include meadows, shrubs, coniferous forests, and broadleaf forests. With the terrain high in the south and low in the north, the elevation varies from <2000 m above sea level (a.s.l.) to around 4900 m a.s.l (Figure 2A). The mountains are classified into three categories: high- (≥4000 m), high- and medium- (3000 m to 4000 m), and medium- (≤3000 m) elevation mountains [23], which contribute to the vegetation stratification. The primeval forest in the park covers an area of 430 km^2^ and occupies 63.5% of the park. Therefore, there is a buffer zone with an area of 600 km^2^ for ecosystem diversity protection in the park (Figure 2B). In addition, the climate here is monsoon dominated and semi-humid with sufficient rainfall, which is very conducive to the growth of precious vegetation, such as *Kingdonia uniflora*, *Ginkgo biloba*, and *Taxus chinensis*. However, several fractures run through the study region, such as the Tazang Tectonic Belt and Huya Fracture, resulting in undulating topography, staggered valleys, and steep slopes that are easily prone to geological hazards during the rainy season from May to September. For example, four debris flows and several coseismic landslides occurred from 14 September to 25 September 2017 after the Jiuzhaigou earthquake, and these events affected nearly 130 km^2^ of the forest ecosystem [24].

#### 2.1.2. Earthquake-Induced Geohazards

**Figure 3:**
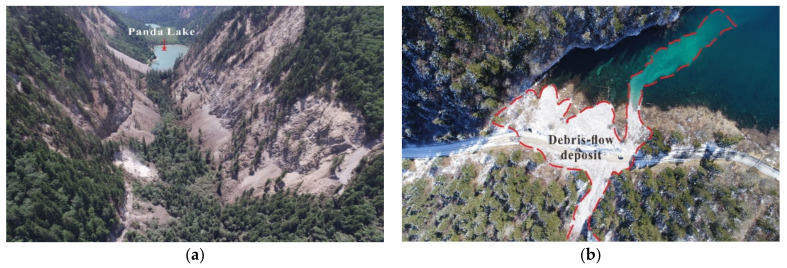
Geohazards caused by the Jiuzhaigou earthquake and their impact on the vegetation. (**a**) Panda Lake and coseismic landslides; (**b**) Xiajijiehai Lake and debris-flow deposit.

In history, fifteen strong earthquakes (M_s_ ≥ 6.0) occurred along the N-S-trending Minjiang faults [24], five of which exceeded M_s_7.0. Since Jiuzhaigou park is far from the epicenters, it was hardly influenced by the five strong earthquakes. However, the Jiuzhaigou earthquake induced landscape fragmentation and frequent geohazards. For example, one of the natural landscape heritage sites, Panda Lake, was seriously damaged by the earthquake-induced geohazards, with vegetation destruction (Figure 3a).

In addition to the direct destruction of vegetation by landslides, debris flow poses an indirect threat to vegetation by inducing coseismic landslides. Three debris flow events occurred in Xiajijiehai Gully shortly after the Jiuzhaigou earthquake on 9, 14, and 25 September 2017, which severely damaged the road, vegetation, and water body (Figure 3b). By remote-sensing and field surveys after the earthquake, 21 earthquake-induced landslides, 84 collapses, and 23 potential debris-flow gullies were identified, respectively. Hu et al. [6] indicated that approximately 30% of the ecosystem in the park, including forests, shrubs, and suitable land for forests, was seriously affected by the Jiuzhaigou earthquake. In some areas, after geohazards, the stability of dam reservoirs may be destroyed, and they may gradually evolve into new terrain, such as barrier dams, under certain conditions [25].

### 2.2. Methodology

#### 2.2.1. Related Data and Preprocessing

The CASA model was adopted to estimate the NPP change considering the diversity of data (NDVI data, as well as earthquake, geohazards, climate, vegetation, and elevation data). Static vegetation parameters [26], reclassified land cover data, and meteorological data were equally indispensable for estimation.

**Figure 4:**
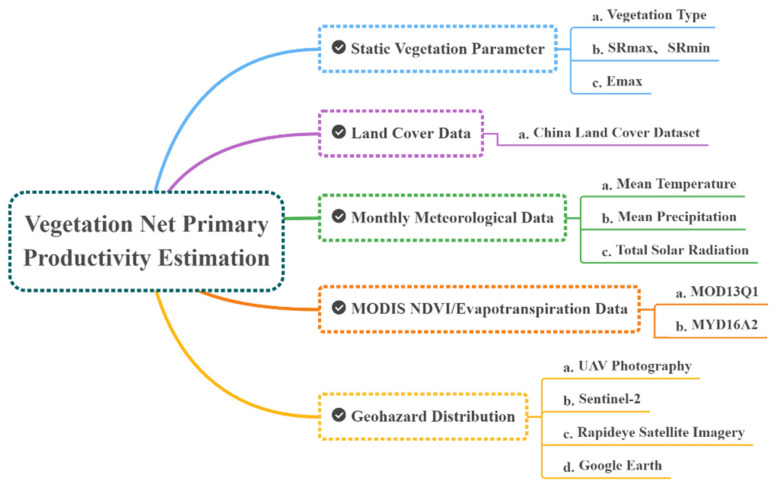
The data components, attributes and sources of NPP estimation.

Several multi-temporal high-resolution remote sensing images, including unmanned aerial vehicle (UAV) photography, Sentinel-2, Rapideye satellite imagery, and Google Earth history imagery (Landsat series), were acquired to identify the earthquake-induced geohazards. DEM data interpolated from large-scale contour maps were obtained from a previous study [24], and the climate, vegetation, and NDVI data were downloaded from NASA and the Environmental and Ecological Science Data Center, among others (Figure 4).

The preprocessing is necessary to fill missing data (lack of certain attribute values), deal with inconsistency (differences in raster), and eliminate noise (errors or outliers) on account of the heterogeneous datasets. The land cover data were obtained from China Land Cover Dataset (CLCD) (https://zenodo.org/record/4417810 [27] accessed on 26 October 2022) for 30 m spatial resolution. The monthly mean temperature data, 20–20 h precipitation data, and monthly total radiation data were taken as meteorological data from 49 weather stations in nine surrounding provinces such as Sichuan, Gansu, Qinghai, and Shanxi, and were obtained from the China Meteorological Data Service Center (http://data.cma.cn/ accessed on 25 September 2020). The NDVI data derived from the MODIS data can be downloaded from the Level-1 and Atmosphere Archive & Distribution System Distributed Active Archive Center (LAADS DAAC) with a spatial and temporal resolution of 250 m and 16 days (MOD13Q1) (https://ladsweb.modaps.eosdis.nasa.gov/search/ accessed on 25 October 2022). Multi-temporal data were obtained in July, August and September from 2015 to 2019 with view of the earthquake’s occurrence. To obtain the data of earthquake-induced geohazards, the satellite imageries were processed to eliminate interference from clouds, atmosphere, and solar elevation angle, among others; UAV remote sensing images were generated and calibrated. In order to intuitively analyze the five-year spatial-temporal change of vegetation NPP in the region struck by the Jiuzhaigou earthquake, the data in the Albers coordinates need to be Kriging-interpolated, extracted, and modified into the same element size (250 m spatial resolution). Since the methods of acquiring these data were described, they will not be specifically covered here.

#### 2.2.2. NPP Estimated by the CASA Model

The CASA model was a process-based remote sensing model driven by the global climate, radiation, soil, and vegetation index datasets. The model was not only suitable for simulating potential vegetation distribution and estimating NPP change but also appropriate for analyzing the relationship between them [17]. In this research, the CASA model proposed by Potter et al. [28] was adopted. The vegetation NPP was mainly determined by the absorbed photosynthetically active radiation (APAR, MJ/m^2^) and the light energy utilization efficiency (Ε, gC/MJ), which were as follows:

(1) $$NPP\left(x,t\right)=APAR\left(x,t\right)\cdot Ε\left(x,\;t\right)$$

(2) $$APAR\left(x,t\right)=0.5\cdot SOL\left(x,t\right)\cdot FPAR\left(x,\;t\right)$$

(3) $$Ε\left(x,t\right)=T_{E1}\left(x,t\right)\cdot T_{E2}\left(x,t\right)\cdot W_{E}\left(x,t\right)\cdot {Ε}_{max}$$

(4) $$FPAR\left(x,\;t\right)=min\left\{\left[SR\left(x,t\right)-SR_{min}\right]/\left(SR_{max}-SR_{min}\right),0.95\right\}$$

(5) $$SR\left(x,t\right)=\left[1+NDVI\left(x,t\right)\right]/\left[1-NDVI\left(x,t\right)\right]$$

(6) $$T_{E1}\left(x,t\right)=0.8+0.02\cdot T_{opt}\left(x\right)-0.0005\cdot {\left[T_{opt}\left(x\right)\right]}^{2}$$

(7) $$T_{E2}\left(x,t\right)=\frac{1.184}{1+e^{0.2\times \left[T_{opt}\left(x\right)-10-T\left(x,t\right)\right]}}\cdot \frac{1}{1+e^{0.3\times \left[-T_{opt}\left(x\right)-10+T\left(x,t\right)\right]}}$$

(8) $$W_{E}\left(x,t\right)=0.5+0.5\cdot ET\left(x,t\right)/ET_{p}\left(x,t\right)$$

where *x*, *t* represent the pixel and month, respectively; *SOL*(*x*,*t*) represents the total solar radiation of pixel *x* in *t* month obtained from meteorological data; *FPAR* represents the fraction of photosynthetically active radiation, defining a linear function of *NDVI*; *SR*, transformation of *NDVI*, represents the simple ratio, and the value of *SR_max_* is determined by vegetation type and is related to *SR* when all downwelling solar radiation is intercepted [15], while *SR_min_* represents the *SR* of unvegetated land areas remaining at 1.08 [26]; *T_opt_*(*x*,*t*) (°C) represents the optimum temperature for vegetation growth during the month of *NDVI_max_* at a certain extent; *T_E1_*(*x*,*t*) (°C) and *T_E2_*(*x*,*t*) (°C) denote the stress function of low temperature and high temperature to light energy utilization, respectively, among which *T_E1_*(*x*,*t*) means the limitation of photosynthesis caused by plants’ biochemical effects at high and low temperatures, while *T_E2_*(*x*,*t*) shows the decreasing trend of vegetation light energy utilization efficiency from *T*_opt_ to critical temperature; *T*(*x*,*t*) is the mean temperature of pixel *x* in *t* month; *W_E_*(*x*,*t*) denotes the water stress coefficient varying from 0.5 to 1 that reflects the effect of available water conditions on light energy utilization efficiency (*E*); *ET* (mm) and *ET_p_* (mm) represent estimated evapotranspiration and potential evapotranspiration obtained from MYD16A2 that are resampled into 1 km spatial resolution; *Ε_max_* (gC/MJ) means the largest light energy utilization efficiency under ideal conditions proposed by Field et al. [29]. As Southwest China is in the high cold-altitude zone, the relatively recognized data (Table 1) obtained by Zhu et al. [26] were selected to better reflect the environmental conditions in the study region. 

#### 2.2.3. Quantitative Impact Calculation

A function of vulnerability and resilience of the sites where earthquake-induced geohazards occurred could be applied for NPP assessment. Thywissen [30] listed various definitions for vulnerability and resilience [6]. In this paper, we propose a new assessment indicator, with vulnerability (*S*) and resilience (*R*) representing the degree of vegetation damage degree and restoration, which could be quantified by NPP reduction during the earthquake and the ratio of NPP increment in 2018 to dur-earthquake level, respectively. In addition, taking into consideration the advocation of the Jiuzhaigou Administration that “ecosystem natural restoration as the primary, manual intervention as an adjunct”, without considering the artificial intervention and hemeroby index mentioned by Winter et al. [31] and Zinnen et al. [32], the impact coefficient (*I*) of quake-induced geohazards on vegetation without self-recovering was proposed, and it can be calculated by Formula (9).

(9) $$I=S\cdot \left(1-R\right)$$

Scholars have shown that NPP change was mainly attributed to natural factors such as temperature and precipitation [33,34,35], which could be regarded as long-term factors in a certain aspect. Nevertheless, the M_s_7.0 earthquake caused instantaneous damage to the ecosystem and was devastating. Therefore, this research principally analyzed the driving factor that affected NPP in a short time, namely seismic intensity (*SI*). The *SI* could be determined by referring to “the distribution map of the M_s_7.0 Jiuzhaigou earthquake and the characteristics of the earthquake damage” released by the Sichuan Earthquake Administration, from which we knew that the seismic intensity in the whole study region is VII+.

Vegetation NPP changes (calculated by Formula (10)) within one month after the earthquake were mainly attributed to earthquake damage, seasonal turnover, and self-restoration, among which earthquake damage and seasonal turnover had negative effects on NPP, while self-restoration exerted positive effects. Thus, we reclassified the NPP change into positive part (ΔNPP>0) and negative part (ΔNPP<0) so that we could determine whether the vegetation was recovered or destroyed in the study region. Then, the value of NPP change was normalized (Formula (11)).

(10) $$\Delta NPP\left(x\right)=NPP^{*}\left(x\right)-NPP^{**}\left(x\right)$$

(11) $$N^{*}\left(x\right)=\left[\Delta NPP\left(x\right)-N_{min}\right]/\left[N_{max}-N_{min}\right]$$

where Δ*NPP*(*x*), NPP*(*x*) and NPP**(*x*) are the NPP change values of pixel *x* during the month when the earthquake occurred, in the month after the earthquake and in the month before the earthquake, respectively; N*(*x*) is the normalized NPP change value of pixel *x*; *N_max_* and *N_min_* are the maximum and minimum value of NPP change within the whole region.

(12) $$S\;\left(\mathrm{or}\;R\right)=\sum_{i=1}^{5}\left(A_{i}\times B_{i}\right)$$

The computed normalized values were divided into 0–0.2, 0.2–0.4, 0.4–0.6, 0.6–0.8, 0.8–1 (5 groups), with the gradient being 0.2, and then the area proportion of each group was calculated. Due to the principle of class mid-value, the median value (0.1, 0.3, 0.5, 0.7, 0.9) was taken as coefficient *A*, and the corresponding area proportion value of each group was regarded as coefficient *B*. Subsequently, based on the method of calculating the expected value, the sum of *A* times *B* was defined as the vulnerability index *S* (Formula (12)). The calculating method for the resilience index *R* was almost the same as that for *S*, but there were differences in the month selection. 

## 3. Results and Analyses

### 3.1. Distribution and Characteristics of NPP

**Figure 5:**
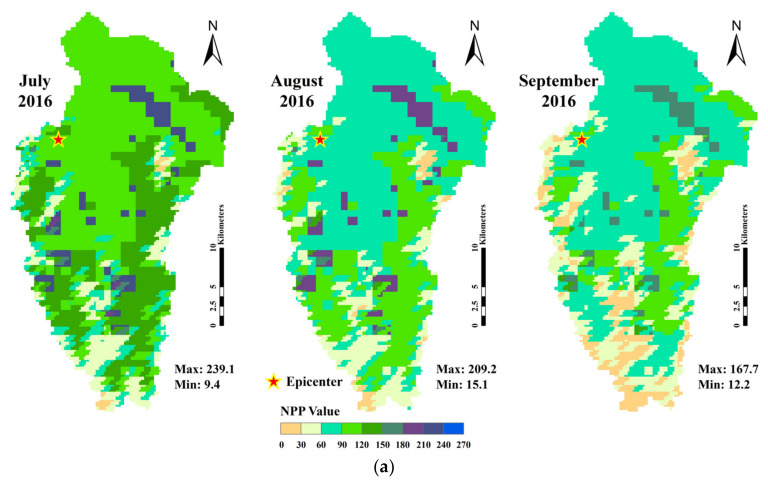

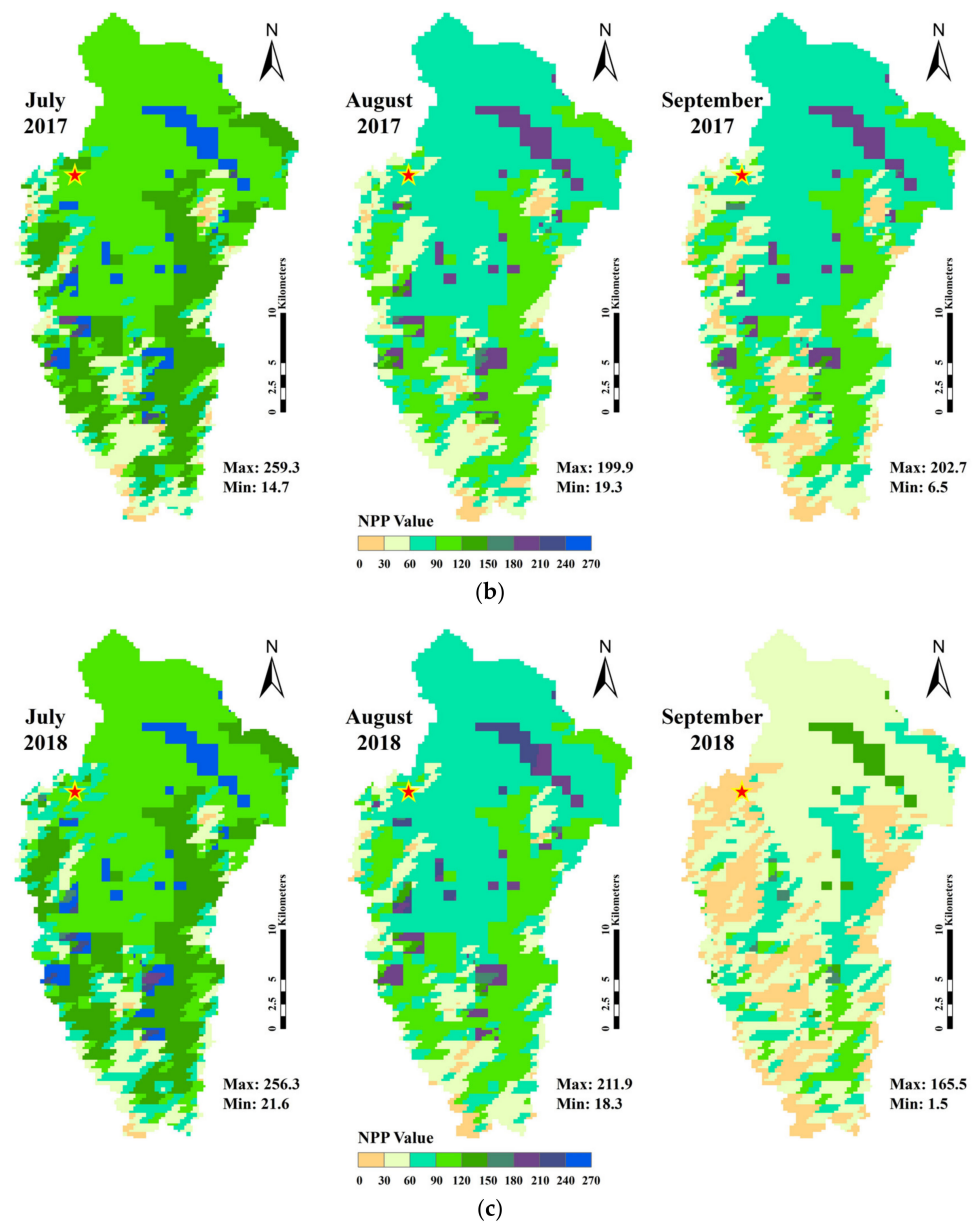
The overview of NPP value within the study region in 2016 (**a**), 2017 (**b**) and 2018 (**c**) (the unit of NPP value was unified as gC/m^2^).

Corresponding data were imported into the CASA model to estimate the NPP value of the study region, and values in July, August and September of 2016, 2017 and 2018 are displayed in Figure 5. In accordance with the value range (upper limit close to 270 gC/m^2^) and study period, the data, each of which equally accounted for 30 gC/m^2^, were divided into nine groups for refining values of pixels.

From Figure 5, the NPP generally decreased from July to September in each year, which might be related to the season. In August 2017, a complex variation of the color block was found in the western part of the study region, and the changes of NPP values near the epicenter were larger. One month after the earthquake, the color block in the central part and western part revealed a decline on NPP, and although the vegetation was recovering, the season was still the dominant factor. Besides, the monthly NPP changes in areas around the epicenter (central part and northern part) were relatively uniform, and the changes in areas far away from the epicenter were complex. The largest NPP ranges were found in the northeastern part and western part of the study region. From the perspective of elevation, the southern region with an altitude higher than 3000 m had diverse terrain that might affect the vegetation NPP to varying degrees when the earthquake occurred. The highest NPP value was found in the area at the altitude of 3000–4000 m, and the lowest NPP value was found in the area above 4000 m.

**Figure 6:**
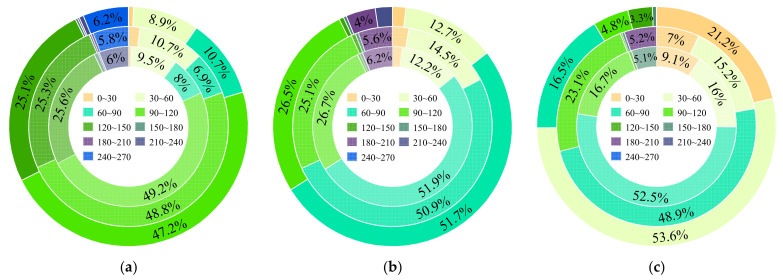
The area proportions of NPP value. The small, medium, and large annuli denote area proportions in 2016, 2017 and 2018, respectively. (The proportion less than 3.0% was not marked. The transparency of the same attribute color decreases by 20% from the inner annulus to the outer annulus). (**a**) July; (**b**) August; (**c**) September.

As the NPP in the west and south of the study region changed significantly during the earthquake and had a broad range within adjacent pixels that existed in the high-altitude area, the area proportion represented by vegetation NPP of the whole study region was significant for evaluating the ecosystem of Jiuzhaigou Valley. Therefore, the NPP values were classified based on the number of pixels, and the area proportions are shown in Figure 6.

From Figure 6a, the areas with NPP value of 90–120 gC/m^2^ accounted for nearly 50% of the whole study region, and the maximum value range was found in July 2017 and 2018. In addition, the NPP level in July 2018 is equivalent to that before the earthquake, indicating that the ecosystem was recovering continuously after the earthquake. The NPP value of more than 70% of the study region was 90–150 gC/m^2^, which was consistent with the finding of Zhang et al. [36]. In August (Figure 6b), the NPP value decreased, and the area with lower NPP values became larger. The NPP values of most areas were 60–150 gC/m^2^, and the color block indicating the maximum range did not appear. However, a larger value range appeared in August the next year, which proved that grasslands and arbor forests with higher productivity showed stronger resilience [36]. In September (Figure 6c), the temperature gradually decreased, and the NPP value changed greatly. One month after the earthquake, the value range peaked, while the value was smaller than 60 gC/m^2^ in September 2018.

NPP_sum_ represented the total NPP value of the vegetation ecosystem. Owing to the diversity of the ecological system, NPP_sum_ values are comprised of maximum (NPP_max_) and minimum values in the region according to zonal analysis. The NPP_max_ value was taken to reflect the maximum intensity of vegetation productivity. Moreover, NPP_mean_, as the average value of the ecological productivity quality, was used to reflect the overall productivity level of plants within a specified zone. The NPP_max_ and NPP_mean_ values from 2015 to 2019 are shown in Table 2.

From Table 2, the NPP_max_ showed an overall decreasing trend with seasonal change in each year. During the earthquake, the NPP reached the lowest value of 199.9 gC/m^2^ and only showed an increase of 2.8 gC/m^2^ during the month after the earthquake, which was attributed to the aftershocks. The NPP_mean_ value was around 110.1 gC/m^2^ in July, 98.4 gC/m^2^ in August and 71.9 gC/m^2^ in September. Its variation was similar to that of NPP_max_. The NPP_mean_ value, less affected by earthquake-induced geological disasters, also quantitatively represented the damage and restoration of vegetation before and after the earthquake and can reveal the change law of vegetation NPP. Compared with the NPP values in 2015 and 2016, those in the same month in 2018 and 2019 were higher, and the integral decline decreased year by year. It is indicated that the vegetation was reconstructed by natural restoration (restoration of soil fertility and species diversity) and artificial intervention (flood control, afforestation and reducing human engineering activities) [37], which could restore the productivity of forest-grass-shrub ecosystems and promote ecological restoration. Furthermore, the NPP_max_ value reached the highest level (261.9 gC/m^2^) in August 2019, suggesting that the ecosystems were rebuilt. The NPP values show an overall downward trend and then an upward tendency.

### 3.2. NPP Change Attributed to Geohazards

**Figure 7:**
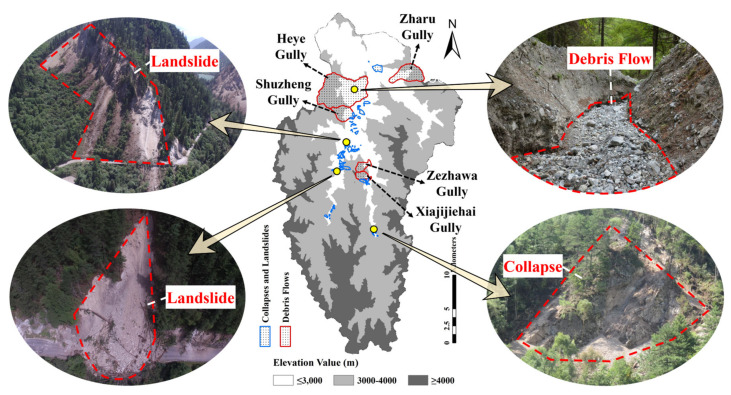
The areas affected by geohazards, including landslides, debris flows and collapses and the scenes of typical earthquake-induced geohazards in Jiuzhaigou Valley.

Geohazards in gullies and valleys can further cause vegetation destruction. Geohazards are classified in terms of their causes. Landslide is attributed to the instability of rock-soil mass of a high-steep slope and overall sliding along the weak surface. Debris flow is caused by heavy rainfall, after which the slope runoff carries the loose soil with large pores and gradually converges into a flood with fast speed and large flow. Collapse is a rolling phenomenon that often occurs on steep slopes or reverse slopes. Based on the classification above, 128 geohazards identified by field survey that caused damage to vegetation were divided into landslides, collapses and debris flows.In light of the area where geohazards occurred, landslide and collapse were integrated, and debris flows mainly occurred in Shuzheng Gully, Zharu Gully, Heye Gully, Zezhawa Gully and Xiajijiehai Gully. By combining the DEM data, the areas affected by representative geohazards are shown in Figure 7.

The study shows that the area affected by debris flow is larger than those affected by collapse and coseismic landslide. According to the land cover database, the land cover type in five debris-flow gullies in the study region was mostly needle-leaved evergreen forest with a small proportion of alpine and sub-alpine meadow and plain grassland in Zharu Gully. The land cover type in areas affected by collapse and coseismic landslide was the needle-leaved evergreen forest. In addition, the land cover type in the central and northern areas was needle-leaved evergreen forest, where the NPP value in the same year shows a rising tendency with time.

**Figure 8:**
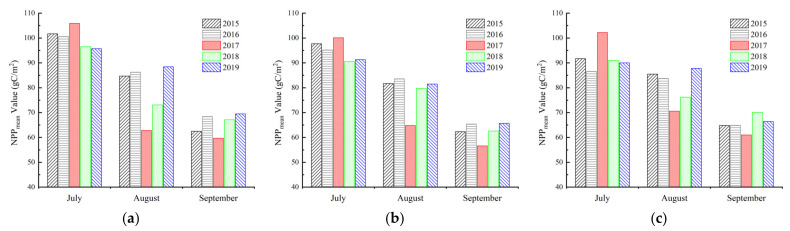
The NPP_mean_ change caused by the three earthquake-induced geohazards in 2015 to 2019. (**a**) Landslide; (**b**) Debris flow; (**c**) Collapse.

The mensurable change of NPP in geohazard zones directly manifested the influence of the earthquake on these zones. Therefore, the law of NPP_mean_ change caused by the three representative geohazards from 2015 to 2019 was analyzed (Figure 8).

From Figure 8, the NPP_mean_ values in areas hit by landslides, debris flows, and collapses were 59.7–105.9 gC/m^2^, 56.6–100.1 gC/m^2^, and 63.7–100.2 gC/m^2^, respectively. Before the earthquake, the NPP_mean_ values in geohazard zones in the same month had little difference (within 10 gC/m^2^). Additionally, the NPP_mean_ values of landslide and collapse zones were generally higher than the values of debris flow zones. During the earthquake, the values sharply declined by 27.2% (landslide), 22.4% (debris flow) and 15.7% (collapse) compared with those in the same month in 2016, and the largest decline was caused by landslides. After the earthquake, the NPP_mean_ values began to rebound and vegetation generally recovered, and the values became close again during the study period. The vegetation in debris flow zones showed a stronger recovery than the vegetation in landslide and collapse zones, with a maximum NPP_mean_ increase of about 23.0% compared with the corresponding value in August 2017. Through the analysis of the above results, it could be preliminarily concluded that, to some extent, geohazards had a negative correlation with vegetation NPP change.

### 3.3. Vegetation-Earthquake Impact Assessment

In accordance with the designed assessment system, the quantitative change of NPP before and after the earthquake was calculated by Formula (10). After normalization (Formula (11)), the Δ*NPP* values corresponding to each seismic intensity were obtained, and vulnerability indices were calculated, as shown in Table 3.

From Table 3, the resilience (*R*) one month after the earthquake was 0.642–0.693, and the resilience (*R*) of the whole study region was 0.671. At an intuitive ecological level, resilience was determined by the natural restoration of the ecosystem, and the results had a high referential value. However, the vulnerability (*S*) was 0.470–0.669, with the range slightly broader than that of resilience, but the vulnerability (*S*) of the whole study region was 0.473 and was within the normal range. The impact coefficient (*I*) calculated by Formula (9) was 0.146–0.213. Also, the vulnerability and resilience indices corresponding to a single seismic intensity range remained similar, and the indices corresponding to the IX+ intensity were markedly different from those corresponding to other intensity ranges. In fact, the seismic intensity was affected by the epicentral distance and the smaller the epicentral distance, the greater the intensity [38]. The magnitude (7.0) of the Jiuzhaigou earthquake could be converted into an intensity of more than 10 by a correlation formula [39,40], and Sichuan Earthquake Administration did not release the map of seismic intensity above 9. Therefore, it is reasonable to think that the specificity of the index is affected by the uncertain intensity boundary range.

In the process of studying the damage degree and recovery level of vegetation before and after the earthquake, it was found that almost all (98.98%) vegetation in IX+ seismic intensity zones was damaged; the vegetation in VIII–IX seismic intensity zones was slightly less damaged (94.84%); VII–VIII seismic intensity had the least impact on NPP (82.79%). Vegetation in IX+ seismic intensity zones recovered best (93.12%) (Table 4). It could be preliminarily concluded that the higher the seismic intensity, the greater the damage degree and recovery level.

## 4. Discussion

The possible reason why landslides and collapses cause more serious damage is that large plants with greater productivity are more able to grow in areas with relatively dense soil structures. As to the stronger recovery of vegetation in debris flow zones, by checking the relevant land cover database, it was found that the major vegetation types in the five debris-flow gullies are shrub and grassland [27]. It is difficult for large plants to take root in a short time in areas where the soil was loosened after debris flow [41], while herbs and shrubs with short growth cycles could grow rapidly in the debris flow regions. Previous geological and topographical studies provide a theoretical basis for the conclusion of this research. The Jiuzhaigou earthquake-induced debris flow and collapse were characterized by short duration and rapid occurrence [24]. In contrast, the coseismic landslide was related to the seismic susceptibility of ground surface and valley morphology affected by paleo-glacier and river incision [42]; the affected area was large, and the duration was long. Hence, to some extent, it could be preliminarily assumed that landslides caused greater damage to vegetation, and vegetation in debris flow zones showed a stronger recovery after the quake.

In recent years, little attention was paid to the relationship between NPP and earthquakes. However, as an indicator of respiration and photosynthesis of ecological vegetation, vegetation gross primary productivity (GPP), comprised of autotrophic respiration of plants and NPP, was combined with MODIS data, and it was found that the M_s_8.0 Wenchuan earthquake in 2008 caused enormous damage to vegetation and GPP of the severely afflicted area dropped by 22% but then recovered [43]. Nine years later, the M_s_7.0 Jiuzhaigou earthquake triggered various geohazards, which destroyed ground surface plants and decreased NPP value by around 23%. Fortunately, the vegetation in debris-flow gullies and landslide-and-collapse areas after the earthquake was restored despite the aftershocks, and the NPP value returned to a nearly normal level after one year. Furthermore, as a strong dynamic indicator of terrestrial vegetation coverage [44], NDVI also reflected the changing laws of vegetation before and after the earthquake and was similar to NPP to a certain extent. During the Wenchuan earthquake, the NDVI value showed a sharp decrease in areas with a previously high vegetation coverage, which was attributed to emergency events, natural succession or human activities [45]; GPP and leaf area index (LAI) (a crucial parameter of plant photosynthetic model) fluctuated and showed an overall upward trend, and the diversity of plant species was markedly increased correspondingly [46]. From the perspective of intuitive representation, the degree of vegetation destruction was mainly determined by the scale of geohazards. During the natural calamity, the ecosystem showed massive allogenic succession besides autogenic succession. After the geological environment tends to be stable, vegetation began to recover, and the photosynthetic efficiency affected by phenology (e.g., temperature, precipitation, solar radiation) was increased [15,47]; the high enzyme activity promoted vegetation root growth [48] and the differentiation of stem and leaves. As time goes by, vegetation was restored to its original level, and brand-new tourist attractions like Shuanglonghai Waterfall were constructed by human intervention. Despite the success in vegetation restoration and human intervention, WHSs still need to be better protected against potential geohazards.

With the NPP estimation method adopted in this research, the impact of the earthquake was considered, various types of subsequent hazards and their influencing areas were identified, and combined with the CASA model, the impact of the earthquake on NPP change could be preferably analyzed. However, there are some limitations concerning data precision. The maximum composition of 16-day MODIS NDVI data was synthesized from two sets of original NDVI data of one month, and the final NDVI data of one month was synthesized from two sets of original NDVI data of that month, which cannot accurately represent the NDVI data at the time of the earthquake. As to the interpolated meteorological data, the smooth operation of the Kriging method requires quantities of data, which avoid the problem that the semi-variogram could not be estimated. In this paper, *SR_max_* and *Ε_max_* required in this model were determined in the research of Zhu et al. [26], where the data were considered as a recognized standard for applicability in China [49,50]. But when it came to NPP calculation in temperate or subtropical zones, the uncertainties might increase, and therefore, proper adjustments of some indices are needed. Besides, the model precision was associated with many parameters. Based on the accessible remote sensing data with high accuracy, if we could conduct field surveys to further specify the vegetation types in the study region, the model outputs would be closer to real values. In the case of the large study region, the model precision partly depends on the imported land cover data and other surface and upper-air data.

Despite the limitations, this research was valuable as it quantitatively assesses the impact of earthquake-induced geohazards on NPP. Future research could explore more factors, such as NDVI influencing vegetation vitality and NDWI (normalized difference water index) influencing vegetation water content [51]. Finally, in this paper, we built a relationship framework to preliminarily study the impact of geohazards on NPP, and the assessment of the quantitative relationship between NPP and geohazards was the next step. In future research, the data such as temperature, precipitation, and solar radiation will be imported into the CASA model, and the quantitative law of NPP change affected by geohazards with different damage levels will be better understood if geohazard indicators are directly integrated into the CASA model. More detailed data validation will be conducted to verify the specific relationship. By incorporating them into the CASA model, the ecosystem productivity under the influence of geohazards would be evaluated in a diversified way.

## 5. Conclusions

From an ecological and geographical perspective, this study combined the NPP value of seismic areas obtained by the CASA model with the spatial distribution data of geohazards collected by field surveys to explore the effect of seismic intensity on vegetation destruction. The conclusions are as follows:

(1) The NPP value of more than 70% area was 90–150 gC/m^2^ in July. In August, the value showed an overall decrease, and the areas with lower values became larger; the NPP value of most areas was 60–150 gC/m^2^.
(2) NPP_max_ and NPP_mean_ were 151.5–261.9 gC/m^2^ and 54.6–116.3 gC/m^2^, respectively, in July, August and September from 2015 to 2019. The vegetation gradually recovered after the earthquake. The NPP value showed an uptrend in the corresponding periods in 2018 and 2019. The integral decline amplitude decreased by years in comparison to that in 2015 and 2016. The decrease slowed down after the earthquake.
(3) During the earthquake, compared with the NPP value in the same month in 2016, the value in areas affected by geohazards sharply declined by 27.2% (landslide), 22.4% (debris flow) and 15.7% (collapse). The vegetation in debris flow zones showed a stronger recovery, with a maximum increase of about 23.0% in September 2017.
(4) In the aspect of the assessment system, the resilience index corresponding to the seismic intensity ranges one month after the earthquake was 0.642–0.693, and that of the whole study region was 0.671. The vulnerability index corresponding to the seismic intensity ranges was 0.470–0.669, and that of the whole study region was 0.473. The impact coefficient defined to represent the impact of the earthquake on NPP was 0.146–0.213.

## Figures and Tables

**Table 1:** The maximum light energy utilization efficiency (*Ε_max_*) of typical vegetation types.

Vegetation Type	*SR_max_*	*Ε_max_*
Needleleaved deciduous forest	6.63	0.485
Needleleaved evergreen forest	4.67	0.389
Broadleaved evergreen forest	5.17	0.985
Broadleaved deciduous forest	6.91	0.692
Bush	4.49	0.429
Sparse woods	4.49	0.542
Alpine and sub-alpine grassland	4.46	0.542
Lake	4.46	0.542
Rock	4.46	0.542

**Table 2:** The estimated values of NPP based on the CASA model in the study region in July, August and September 2015 to 2019.

NPP Value (gC/m^2^)		2015	2016	2017	2018	2019
NPP_max_	July	260.3	239.1	259.3	255.3	231.3
	August	250.9	209.2	199.9	211.9	261.9
	September	151.5	167.7	202.7	165.5	218.2
NPP_mean_	July	116.3	107.0	111.8	112.8	102.4
	August	105.1	92.1	86.2	92.7	115.8
	September	62.8	69.7	83.6	54.6	89.1

**Table 3:** The area proportion for Δ*NPP* value normalization, vulnerability index (*S*) and resilience index (*R*) of 3 seismic intensities and the whole study region from July to August 2017.

**Dur-quake**	**Seismic** **Intensity**	**Area Proportion for Normalized Values (%)**					**Vulnerability Index (*S*)**	**Impact** **Coefficient (*I*)**
		**0–0.2**	**0.2–0.4**	**0.4–0.6**	**0.6–0.8**	**0.8–1**		
	VII–VIII	1.93	15.28	78.99	3.26	0.54	0.470	0.168
	VIII–IX	0.97	12.49	84.98	1.53	0.03	0.474	0.146
	IX+	1.86	3.26	5.21	87.81	1.86	0.669	0.213
	VII–IX+	1.21	13.23	83.53	1.81	0.22	0.473	0.156
**Post-quake**	**Seismic** **Intensity**	**0–0.2**	**0.2–0.4**	**0.4–0.6**	**0.6–0.8**	**0.8–1**	**Resilience** **Index (*R*)**	**Impact** **Coefficient (*I*)**
	VII–VIII	1.20	2.76	24.03	67.70	4.31	0.642	0.168
	VIII–IX	0.03	0.29	5.66	91.33	2.69	0.693	0.146
	IX+	1.02	2.05	3.07	92.47	1.40	0.682	0.213
	VII–IX+	0.39	1.32	13.38	82.23	2.68	0.671	0.156

**Table 4:** The Δ*NPP* area proportion corresponding to three seismic intensities one month before and after the earthquake.

Seismic Intensity	Area Proportion (%)			
	Dur-Quake		Post-Quake	
	Δ*NPP* < 0 gC/m^2^	Δ*NPP* > 0 gC/m^2^	Δ*NPP* < 0 gC/m^2^	Δ*NPP* > 0 gC/m^2^
VII–VIII	82.79	17.21	45.74	54.26
VIII–IX	94.84	5.16	19.78	80.22
IX+	98.98	1.02	6.88	93.12

## Data Availability

The data presented in this study are available on request from the corresponding author.
